# Clinical characteristics and prognostic factors of male breast cancer in China

**DOI:** 10.3389/fonc.2024.1362826

**Published:** 2024-03-08

**Authors:** Han Lei, Baojie Hua, Yingying Mao, Wei Cui, Caiping Mao, Shaoxue Yang, Jiayu Li

**Affiliations:** ^1^ The First Affiliated Hospital of Zhejiang Chinese Medical University (Zhejiang Provincial Hospital of Chinese Medicine), Hangzhou, China; ^2^ Department of Epidemiology, Zhejiang Chinese Medical University School of Public Health, Hangzhou, China; ^3^ Zhejiang Cancer Hospital, Hangzhou Institute of Medicine (HIM), Chinese Academy of Sciences, Hangzhou, China

**Keywords:** male breast cancer, prognosis, nomogram, overall survival, disease-free survival

## Abstract

**Purpose:**

This study aimed to explore the clinical characteristics of male breast cancer (MBC) patients and the factors influencing their prognosis.

**Methods:**

We conducted a retrospective case series analysis of 117 MBC cases who were treated at Zhejiang Cancer Hospital from 2009 to 2022. Cox proportional hazard model was used to identify prognostic factors of MBC. Nomogram was constructed based on these factors, which was further evaluated by C-index and calibration curves.

**Results:**

A total of 115 MBC cases were finally included in our analyses, with median diagnosis age of 59 years. Of these cases, 80.0% were estrogen receptor (ER) positive, 79.2% were progesterone receptor (PR) positive, 48.7% were human epidermal growth factor receptor 2 (HER2) negative, and 42.6% had Ki67 levels higher than 15%. 108 (93.9%) cases underwent radical mastectomy, while only 3 (2.6%) received breast-conserving surgery. The Logrank test suggested that lymphocyte-to-monocyte ratio (LMR) was negatively associated with both overall survival (OS) and disease-free survival (DFS) of MBC, while platelet-to-lymphocyte ratio (PLR) and neutrophil-to-lymphocyte ratio (NLR) were only positively associated with OS (all P-values < 0.05). Multivariate regression analysis showed that age (HR 1.08, 95% CI 1.03-1.13) was significant prognostic factors for OS. Meanwhile, age (HR 1.06, 95% CI 1.02-1.10), histological differentiation grade (poorly differentiated/undifferentiated *vs.* well-differentiated: HR 2.55, 95% CI 1.05-6.17), and TNM stage (IV *vs*. I: HR 31.59, 95% CI 6.01-165.93) were also significant prognostic factors for DFS. Nomograms were developed for DFS, with C-indexes of 0.782, indicating good predictive performance.

**Conclusion:**

Increased age, bigger tumor size, higher TNM stage, and lower histological differentiation grade were associated with poor MBC prognosis, and LMR, PLR, and NLR might be potential predictors for MBC prognosis.

## Introduction

Male breast cancer (MBC) is a rare cancer that comprises less than 1% of all breast cancer cases (1, 2) and less than 1% of all cancers of male. With the development of social economy and the progress of breast cancer diagnosis technology, the global incidence of MBC has been on the rise (3).

Given the infrequency of MBC cases, current clinical practices for diagnosing, treating, and evaluating the prognosis of MBC often rely on female breast cancer protocols, despite physiological differences between men and women (4, 5). For example, a multicenter study found that CDK 4–6 inhibitors, were effective and safe options for men with hormone receptor-positive (HR+) and human epidermal growth factor receptor 2-negative (HER2-) metastatic breast cancer, similar to their effectiveness in female breast cancer (6). Furthermore, MBC is frequently overlooked, and men are inclined to receive diagnoses at later stages of the disease and at more advanced ages than their female counterparts (7, 8). Common risk factors associated with the prognosis of breast cancer, such as age, ethnicity, tumor size, histological differentiation grade, estrogen receptor (ER), progesterone receptor (PR), human epidermal growth factor receptor 2 (HER2) and Ki-67, have been well-recognized (9–12). Inflammatory biomarkers, such as lymphocyte-to-monocyte ratio (LMR), platelet-to-lymphocyte ratio (PLR), and neutrophil-to-lymphocyte ratio (NLR), have been shown to be associated with the prognosis of esophageal, cervical, and lung cancers (13–17). However, evidence on the relationship between these inflammatory biomarkers and female breast cancer was still limited and inconsistent (18–21). More importantly, the study examining the association between these inflammatory biomarkers and the prognosis of MBC was scarce. So we aimed to investigate the prognostic factors of MBC, including clinical features and inflammatory biomarkers, and construct a nomogram for MBC. Our study might provide important clues for clinical prognosis of MBC.

## Materials and methods

### Study participants

A total of 117 MBC cases who were treated at Zhejiang Cancer Hospital from 2009 to 2022 were enrolled in current study. All MBC cases were diagnosed by clinicians, and confirmed by pathological examination. Exclusion criteria included cases readmitted for the same condition, and those who were unable to complete follow-up. Ultimately, 115 MBC cases were included in our analyses.

### Data collection

Basic characteristics, clinical and histopathological features, metastasis status, treatment methods, and inflammatory biomarkers were collected. NLR was defined as the absolute neutrophil count divided by the absolute lymphocyte count. PLR was defined as the absolute platelet count divided by the absolute lymphocyte count. LMR was defined as the absolute lymphocyte count divided by the absolute monocyte count. In addition, both overall survival (OS) and disease-free survival (DFS) were recorded as the two endpoints in current study. OS was defined as the duration between the diagnosis of MBC and death from any cause. DFS, on the other hand, was defined as the duration between surgery and the occurrence of MBC recurrence (whether local, regional, or distant), diagnosis of a second primary MBC, or death from any cause.

### Statistical analysis

All statistical analyses were performed using SPSS Statistics version 25.0 and R version 4.2.2 software. Continuous variables with normal distribution were described using mean ± standard deviation (SD), otherwise median [(interquartile range) (IQR)] was used. Categorical variables were described using frequency and percentage [n (%)]. Kaplan-Meier survival curves were plotted, and survival differences were compared using the Log-Rank test. Univariate Cox proportional hazards regression model was used to identify significant MBC-related factors, which were further included in a multivariable Cox proportional hazards regression model. A nomogram was developed using R packages such as “rms” and “Survival” based on the identified prognostic factors of MBC, using the Bootstrap method (n = 1000). Discrimination of the nomogram was evaluated using the C-index with its 95% confidence interval (CI). A higher C-index value indicates greater accuracy of the model, and value greater than 0.70 generally indicates good discrimination of the model. Calibration plot was used to assess the consistency between the predicted survival rate and the actual survival rate. The closer the curve is to the 45-degree diagonal reference line, the more accurate the calibration of the model. All tests were two-sided, and a *P*-value < 0.05 was considered statistically significant.

## Results

The age at diagnosis of the cases ranged from 20 to 82 years, with a median (IQR) age of 59.0 (16.0) years, mean (SD) age of 58.6(13.5) years. The clinical features of MBC patients were shown in **Table 1**. Among the cases with MBC, 111 (96.5%) were married, 42 (36.5%) had a history of smoking, and 38 (33.0%) reported a history of alcohol consumption. A family history of tumors was observed in 33.9% of cases, with 6 cases specifically having a family history of breast cancer. 54.8% of tumors were located in the left breast, and the histopathological characteristics were primarily composed of tumors with a size of ≥ 2.0 cm (57.4%), invasive type (83.5%), and poorly differentiated histology (42.6%). Lymph node metastasis was present in 42.6% of cases. The molecular subtypes of the tumors were predominantly estrogen receptor (ER) positive (80%), progesterone receptor (PR) positive (79.2%), human epidermal growth factor receptor 2 (HER2) negative (48.7%), and 66(57.4%) patients had Ki67 levels no higher than 15%. Triple-negative (ER negative, PR negative, and HER2 negative) cases comprised only 1.7% of the total, while 4.3% of cases exhibited HER2 overexpression. The median (IQR) values of LMR, PLR and NLR were 3.67 (2.17), 113.20 (66.33), and 2.13 (1.60), respectively.108 (93.9%) cases underwent radical mastectomy, while only 3 (2.6%) received breast-conserving surgery. In addition, among the 13 MBC patients diagnosed with TNM stage of IV, only one of them did not undergo surgery, while the remaining 12 individuals all received radical mastectomy. Among all patients, 63 (54.8%) individuals received at least one of the postoperative adjuvant treatment of radiotherapy, chemotherapy, or hormone therapy. During follow-up period, 85 (73.9%) cases did not experience cancer distant metastasis, while in patients with metastases, the most frequent metastatic site was lungs (12 patients), followed by lymph nodes and bones (both were 10 patients), with 11 of them developing metastases in two or more locations. After a median follow-up time of 78 months, 29 (25.2%) cases passed away during the follow-up period, and 36 (31.3%) cases had a recurrence.

**Table 1:** MBC patient’s clinical characteristics (*N* = 115).

Characteristics	n (%)	Characteristics	n (%)
**Age(years), median (IQR)**	59.0(16.0)	**ER**	
**Age(years), mean (SD)**	58.6(13.5)	Positive	92(80.0)
**Smoking history**		Negative	8(7.0)
Yes	42(36.5)	Unknown	15(13.0)
No	73(63.5)	**PR**	
**Drinking history**		Positive	91(79.2)
Yes	38(33.0)	Negative	9(7.8)
No	77(67.0)	Unknown	15(13.0)
**BMI (kg/m^2^)**		**HER2**	
< 18.5	3(2.6)	Positive	42(36.5)
18.5 – 25.0	82(71.3)	Negative	56(48.7)
≥ 25.0	30(26.1)	Unknown	17(14.8)
**Family history of cancer**		**LMR, median (IQR)**	3.67(2.17)
Yes	39(33.9)	**PLR, median (IQR)**	113.20(66.33)
No	76(66.1)	**NLR, median (IQR)**	2.13(1.60)
**Tumor location**		**Type of surgery**	
Left	63(54.8)	Breast conserving surgery	3 (2.6)
Right	48(41.7)	Radical mastectomy	108 (93.9)
Bilateral	4(3.5)	No surgery	4 (3.5)
**Tumor size (cm)**		**Postoperative treatment**	
< 2.0	49(42.6)	Yes	63 (54.8)
≥ 2.0	66(57.4)	No	52(45.2)
**Differentiation**		**Distant metastasis** ^1^	
Well	20(17.4)	Yes	30 (26.1)
Moderate	31(27.0)	No	85 (73.9)
Poor/undifferentiated	49(42.6)	**Site of distant metastasis**	
Unknown	15(13.0)	No metastasis	85 (73.9)
**TNM stage**		Lung	12 (10.4)
I	28(24.4)	Bone	10 (6.5)
II	38(33.0)	Lymph node	10 (6.5)
III	23(20.0)	Liver	5 (3.2)
IV	13(11.3)	Brain	2 (1.3)
Unknown	13(11.3)	Adrenal gland	1 (0.6)
**Lymph node metastasis**		Others^2^	5 (4.3)
Positive	49(42.6)		
Negative	66(57.4)		
**Ki67**			
≤15%	66(57.4)		
>15%	47(40.9)		
Unknown	2(1.7)		

IQR, interquartile range; SD, standard deviation; BMI, body mass index; ER, estrogen receptor; PR, Progesterone receptor; HRE2, human epidermal growth factor receptor 2; LMR, lymphocyte-to-monocyte ratio; NLR, neutrophil-to-lymphocyte ratio; PLR, platelet-to-lymphocyte ratio.

^1^Distant metastasis occurred during the postoperative follow-up period.

^2^Other site of distant metastasis including kidney, thorax, chest wall, eyes and stomach.

The median survival time of the 115 cases was 146 months, with a 5-year overall survival rate of 76.8% and a 10-year overall survival rate of 66.9%. Univariable Cox regression analysis for prognostic factors of MBC related to OS and DFS was shown in -**Tables 2**, **3** and **Supplementary Tables 1**, **2**. The results showed that 7 factors were associated with worse OS in MBC: age (hazard ratio [HR] 1.11, 95% confidence interval [CI] 1.07-1.16), tumor size (HR 4.42, 95%CI 1.68-11.61), TNM stage (stage IV ***vs.*** I: HR 7.41, 95%CI 2.13-25.73), Ki67 (HR 2.30, 95%CI 1.07-4.92), distant metastasis (HR 4.56, 95%CI 2.17-9.58),PLR (HR 2.83, 95%CI 1.29-6.24), and NLR (HR 3.16, 95%CI 1.39-7.19),while 4 factors were associated with better OS in MBC: ER (HR 0.31, 95%CI 0.11-0.92), PR(HR 0.31, 95%CI 0.10-0.94), type of surgery(radical mastectomy ***vs.*** no surgery: HR 0.17, 95%CI 0.04-0.75), and LMR(HR 0.38, 95%CI 0.17-0.86). As for DFS, there were six factors associated with DFS in MBC: age (HR 1.06. 95%CI 1.02-1.09), tumor size (HR 3.19, 95%CI 1.57-6.47), histological differentiation grade (poorly differentiated/undifferentiated vs. well-differentiated: HR 3.98, 95% CI 1.37-11.55), TNM stage (stage IV vs. I: HR 29.50, 95%CI 7.62-114.18), Ki67 (HR 2.74, 95%CI 1.41-5.35), and LMR (HR 0.50, 95%CI 0.26-0.96). Furthermore, above statistically significant factors were included in multivariate Cox regression analysis. We only observed a positive association between age and OS in MBC (**Table 2**), as well as age, tumor size, histological differentiation grade, and TNM stage with DFS (**Table 3**). Regarding the three inflammatory biomarkers LMR, PLR, and NLR, we plotted their associations with the OS and DFS of MBC using Kaplan-Meier survival curves. The Logrank test revealed that LMR was significantly negatively associated with both OS and DFS of MBC, while PLR and NLR were only positively associated with OS (all P-values < 0.05) (**Supplementary Figures 1A–D**).

**Table 2:** Univariable and multivariate Cox regression analysis for prognostic factors of MBC related to overall survival.

Factors	Univariable Cox regression		Multivariate Cox regression	
	HR (95%CI)	*P*-value	HR (95%CI)	*P*-value
**Age(years)**	1.11(1.07-1.16)	4.347E-7	1.08(1.03-1.13)	0.001
Tumor size(cm)				
< 2.0	1.00(reference)		1.00(reference)	
≥ 2.0	4.42(1.68-11.61)	0.003	2.04(0.63-6.59)	0.233
TNM stage				
I	1.00(reference)		1.00(reference)	
II	2.03(0.62-6.61)	0.240	1.37(0.37-5.05)	0.636
III	3.12(0.96-10.17)	0.059	1.61(0.43-6.00)	0.480
IV	7.41(2.13-25.73)	0.002	2.62(0.54-12.75)	0.233
Ki67				
≤15%	1.00(reference)		1.00(reference)	
>15%	2.30(1.07-4.92)	0.033	0.87(0.33-2.27)	0.769
ER				
Negative	1.00(reference)		1.00(reference)	
Positive	0.31(0.11-0.92)	0.035	0.64(0.09-4.54)	0.659
PR				
Negative	1.00(reference)		1.00(reference)	
Positive	0.31(0.10-0.94)	0.038	0.33(0.05-2.17)	0.247
Type of surgery				
No surgery	1.00(reference)		1.00(reference)	
Breast conserving surgery^1^	0.17(0.01-1.96)	0.154	–	–
Radical mastectomy	0.17(0.04-0.75)	0.019	0.26(0.03-1.94)	0.187
Distant metastasis				
No	1.00(reference)		1.00(reference)	
Yes	4.56(2.17-9.58)	6.200E-5	1.15(0.42-3.14)	0.786
LMR				
< 3.67	1.00(reference)		1.00(reference)	
≥ 3.67	0.38(0.17-0.86)	0.020	1.58(0.45-5.49)	0.474
PLR				
< 113.20	1.00(reference)		1.00(reference)	
≥ 113.20	2.83(1.29-6.24)	0.010	0.82(0.29-2.36)	0.713
NLR				
< 2.13	1.00(reference)		1.00(reference)	
≥ 2.13	3.16(1.39-7.19)	0.006	1.46(0.48-4.42)	0.505

HR, hazard ratio; CI, confidence interval; ER, estrogen receptor; PR, Progesterone receptor; LMR, lymphocyte-to-monocyte ratio; NLR, neutrophil-to-lymphocyte ratio; PLR, platelet-to-lymphocyte ratio.

^1^ In multivariate Cox regression, there was only one patient in this group, so HR (95%CI) and *P*-value could not be calculated.

**Table 3:** Univariable and multivariate Cox regression analysis for prognostic factors of MBC related to disease-free survival^1^.

Factors	Univariable Cox regression		Multivariate Cox regression	
	HR (95%CI)	*P*-value	HR (95%CI)	*P*-value
**Age(years)**	1.06(1.02-1.09)	0.001	1.06(1.02-1.10)	0.007
Tumor size(cm)				
< 2.0	1.00(reference)		1.00(reference)	
≥ 2.0	3.19(1.57-6.47)	0.001	2.55(1.05-6.17)	0.038
Differentiation				
Well	1.00(reference)		1.00(reference)	
Moderate	1.42(0.43-4.73)	0.566	1.16(0.33-4.05)	0.822
Poor/ undifferentiated	3.98(1.37-11.55)	0.011	3.23(1.03-10.12)	0.044
TNM stage				
I	1.00(reference)		1.00(reference)	
II	1.56(0.69-3.53)	0.289	1.84(0.65-5.20)	0.248
III	1.72(0.61-4.84)	0.304	0.87(0.25-2.97)	0.818
IV	29.50(7.62-114.18)	9.499E-7	31.59(6.01-165.93)	4.500E-5
Ki67				
≤15%	1.00(reference)		1.00(reference)	
>15%	2.74(1.41-5.35)	0.003	1.20(0.53-2.73)	0.659
LMR				
< 3.67	1.00(reference)		1.00(reference)	
≥ 3.67	0.50(0.26-0.96)	0.037	0.97(0.44-2.13)	0.933

HR, hazard ratio; CI, confidence interval; PR, progesterone receptor; LMR, lymphocyte-to-monocyte ratio; PLR, platelet-to-lymphocyte ratio.

^1^Only patients who did not develop distant metastases during the follow-up period were retained in this analysis.

**Figure 1:**
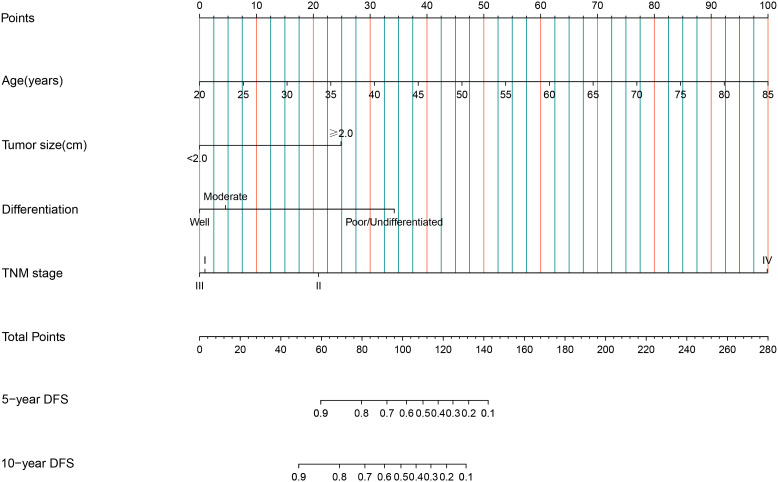
Nomograms of 5-year and 10-year for DFS among MBC cases. Each factor in the nomogram was assigned a weighted number of points, and the total points for each case corresponded to 5-year or 10-year predicted DFS.

**Figure 2:**
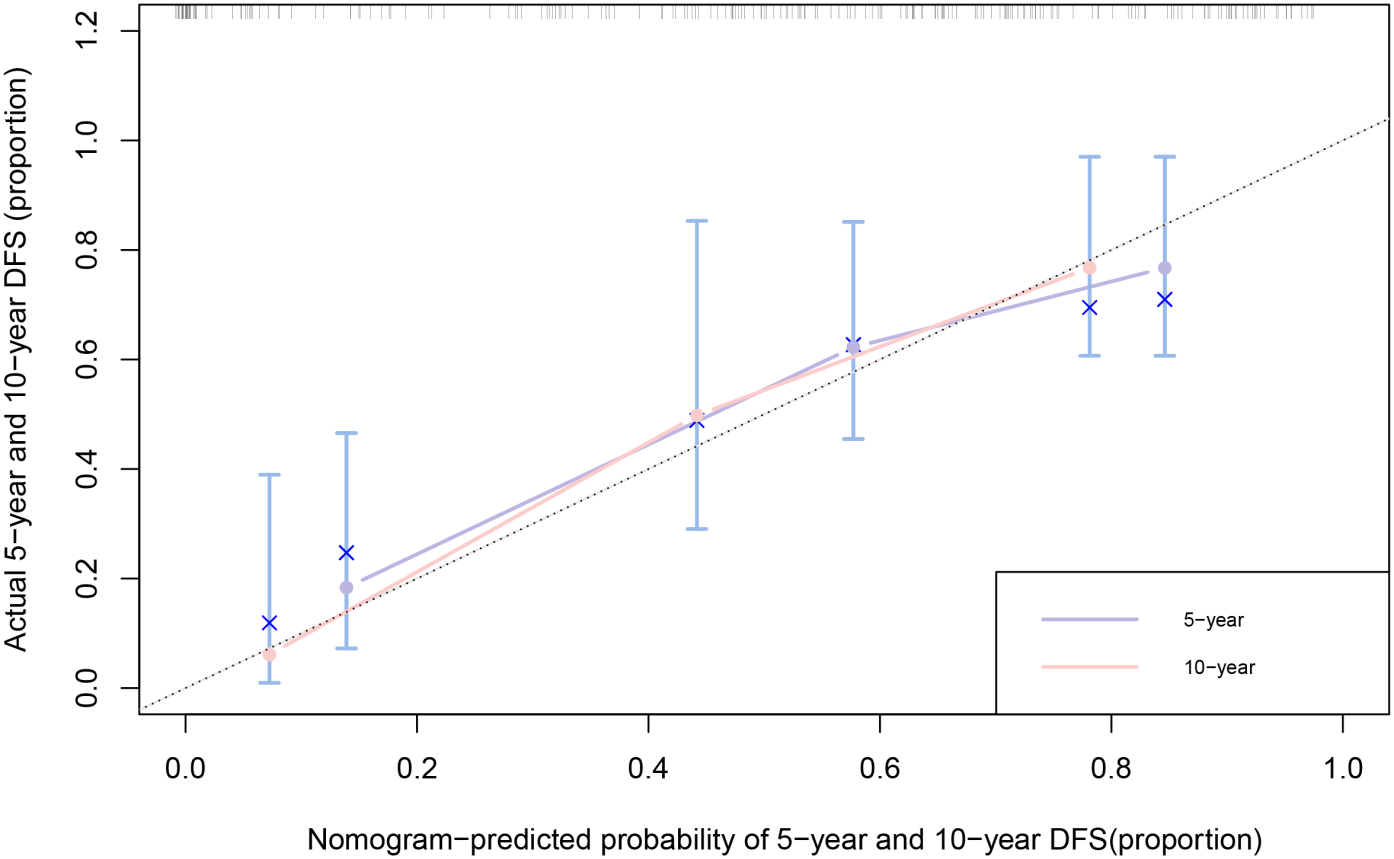
Calibration curves of 5-year and 10-year for DFS among MBC cases. The x-axis is nomogram-predicted probability of survival and y-axis is actual survival. The bootstrapping method was used for the internal validation of the nomogram. The black dotted line indicates perfect calibration.

Nomograms for predicting DFS in MBC cases by incorporating the statistically significant factors in multivariate Cox regression analysis were shown in **Figure 1**. The prognostic nomograms showed good discrimination, with C-index values of 0.782 (95% CI: 0.578-0.904) for DFS. The bootstrapped calibration curves of the nomograms for the predicted *vs.* actual survival probability demonstrated a good fit (**Figure 2**).

## Discussion

Our study identified older age as significant prognostic predictors for both OS and DFS in MBC cases, and poor tumor histological differentiation, bigger tumor size, TNM stage of IV were only found to be associated with a shorter DFS. Although LMR were all significantly associated with both OS and DFS in univariate analysis, and PLR and NLR were only significantly associated with OS, all these three biomarkers did not show statistically significant in multivariate analysis.

The median age of MBC cases in our study was 59 years (range 20-82 years) at diagnosis, which was in line with previous study reporting a similar median age of around 60 years old in MBC cases of different races (9, 22–24). Of the MBC cases in our study, 33.9% had a family history of malignant tumors, including 5.2% with a family history of breast cancer, which is consistent with previous research indicating that 5% to 10% of MBC cases have a family history of cancer (9, 25). Invasive ductal carcinoma was reported to be the most common histopathological subtype of MBC cases (2, 7). Similarly, in our study, 72.2% of MBC cases were diagnosed with invasive ductal carcinoma. In addition, the majority of MBC cases of our study had a TNM stage of I or II, which was consistent with previous research (22, 24). Previous studies have indicated that most MBC patients have ER and PR positive tumors while being HER2 negative (2, 23, 26). In our study, approximately 80% of MBC patients exhibited positive expression of ER and PR, but negative expression of HER2. Previous studies have reported that the primary tumor was commonly found in the left breast (25), and the most frequent sites of distant metastasis were the bone, lung, and lymph nodes (25, 27). Similarly, 54.8% of patients in our study were diagnosed with breast cancer in the left breast, and the three most common sites of metastasis were the bones, lungs, and lymph nodes.

Previous studies have reported inconsistent findings regarding the prognostic factors in MBC cases. A study of 10,873 MBC cases from the National Cancer Data Base in the US showed that older age, black race, higher Charlson comorbidity index, higher tumor grade and stage, and receipt of total mastectomy were associated with poorer OS, while residing in a high income area, positive PR expression and administration of chemotherapy, radiation or endocrine therapy were associated with better OS (22). For immunohistochemistry indicators of MBC, a study based on the SEER database found that MBC cases with HER2-negative having longer OS and higher 4-year OS rates, but did not significantly affect disease-specific survival (DSS) (28). A case-control study involving 65 male breast cancer patients from the Department of Veteran’s Affairs (DVA) Cancer Registry found that the survival rate was higher for ER-positive patients, while PR status and Ki67 were not associated with survival in men with breast cancer (29). A cohort study including 643 MBC cases from Danish found that increased age, bigger tumor size, positive lymph node status, higher grade and Luminal B subtype were risk factors for OS in MBC cases (30). For Chinese population, a study with 152 MBC cases reported that tumor size, radical mastectomy, and hormone therapy were risk factors for both OS and DFS in MBC cases (31), while another study of 77 Chinese MBC cases only found that M stage was significant prognostic factor, and ER, PR, and HER2 status had no impact on OS of MBC (9). In our study, multivariate analysis showed that age were significant prognostic factors for OS of MBC, and age, tumor size, histological differentiation grade, and TNM stage were significant prognostic factors for DFS, and only univariate analysis showed that Ki67>15% was associated with shorter OS and DFS of MBC, while ER and PR positive was associated with longer OS. However, HER2 expression was not significantly associated with either OS or DFS of MBC. Our study’s results might differ from other studies due to differences in ethnicity, sample size, and consideration of confounding factors.

Previous studies have reported inconsistent results regarding the association between inflammatory markers and prognosis of breast cancer. Several studies suggested that higher levels of LMR were associated with better prognosis of breast cancer, while higher levels of PLR were associated with worse prognosis (20, 32–34). However, another study indicated that NLR, PLR, and LMR in MBC had no statistically significant correlation with either DFS or OS (18). The potential mechanisms of LMR, PLR, and NLR might involve that T lymphocytes such as CD4 and CD8 play a role in tumor suppression mechanisms such as cancer immunosurveillance and cancer immunosedition by inducing tumor cell apoptosis, thereby inhibiting tumor cell proliferation and migration (35). Our study reported that higher LMR levels were associated with better prognosis of MBC, and higher PLR and NLR levels were associated with worse prognosis of MBC.

A nomogram is a simple graphical representation of a statistical prediction model that estimates the probability of an event and is widely used in cancer prognosis (36). As the fifth year after surgery is the high-risk period for recurrence and metastasis of breast cancer cases (32), and the 10-year survival rate of breast cancer is over 50% (37), predicting the 5-year and 10-year survival rates of MBC cases is of great clinical significance. Therefore, we constructed nomograms for DFS for MBC cases, with predictors of age, tumor size, histological differentiation grade, and TNM stage. The C-index of nomogram showed a high degree of discrimination (C-index: 0.782, 95%CI: 0.578-0.904), and the calibration curves displayed good accuracy, suggesting that this nomogram can effectively predict the 5-year and 10-year DFS of MBC cases and provide a basis for predicting their prognoses.

There were some limitations in the present study. Firstly, this is a single-center, retrospective study, and some cases’ basic data was not complete, which may lead to bias. Secondly, nomogram model for MBC was only validated internally, and external validation was needed. Thirdly, we were unable to conduct subgroup analyses according to different subtypes or other characteristics due to the limited sample size of MBC cases. Fourthly, previous studies have reported the correlation between breast cancer and genetic factors (5), however, genetic data were not collected in current study.

In conclusion, MBC cases were mainly ER and PR positive, HER2 negative. Age was significant factors influencing OS of MBC, whereas age, tumor size, histological differentiation grade, and TNM stage were significant factors influencing DFS of MBC. Inflammatory markers might hold certain predictive value for the prognosis of MBC. Hence, future clinical practice needs to allocate appropriate attention to inflammation biomarkers, while larger sample studies are warranted to further verify our findings.

## Data availability statement

The original contributions presented in the study are included in the article/**Supplementary Material**. Further inquiries can be directed to the corresponding authors.

## Ethics statement

The studies involving humans were approved by the Ethical Committee of Zhejiang Cancer Hospital (No.IRB-2020-237). The studies were conducted in accordance with the local legislation and institutional requirements. The participants provided their written informed consent to participate in this study.

## Author contributions

HL: Investigation, Resources, Software, Writing – original draft. BH: Conceptualization, Software, Visualization, Writing – original draft. YM: Validation, Writing – review & editing. WC: Investigation, Resources, Writing – review & editing. CM: Investigation, Resources, Writing – review & editing. SY: Methodology, Supervision, Writing – review & editing, Funding acquisition. JL: Methodology, Supervision, Writing – review & editing.

## Supplementary material

Supplementary Figure 1 Kaplan-Meier analyses for OS **(A–C)** and DFS **(D–F)** in MBC cases according to lymphocyte-to-monocyte ratio (LMR), platelet-to-lymphocyte ratio (PLR), and neutrophil-to-lymphocyte ratio (NLR).
