# Supplementary figures and images for: Clinical characteristics and prognostic factors of male breast cancer in China
Source: Front Oncol. 2024 Mar 8;14:1362826. doi: 10.3389/fonc.2024.1362826 (PMC10957788; doi:10.3389/fonc.2024.1362826)

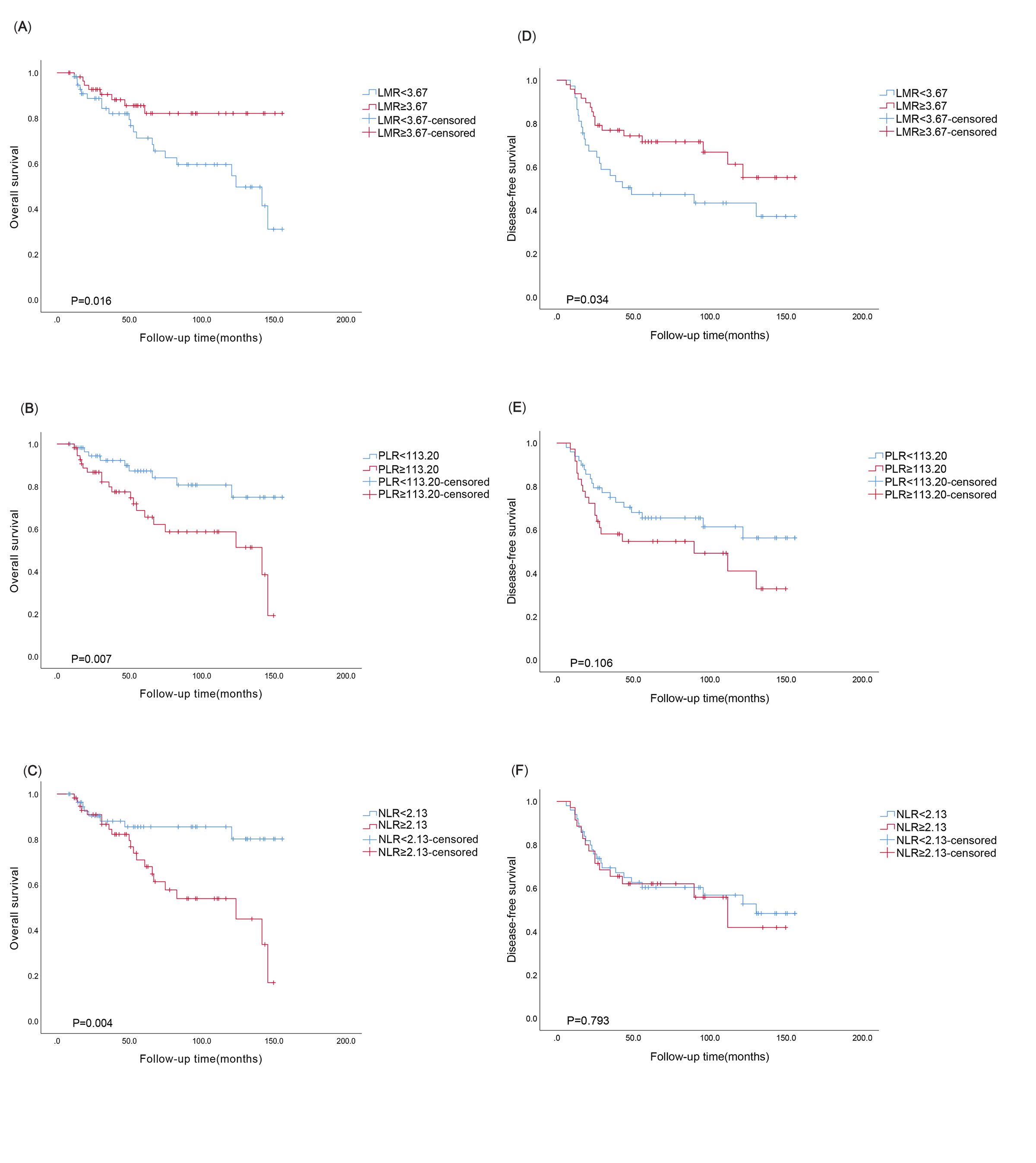

Supplement: Supplementary Figure 1 — Kaplan-Meier analyses for OS (A–C) and DFS (D–F) in MBC cases according to lymphocyte-to-monocyte ratio (LMR), platelet-to-lymphocyte ratio (PLR), and neutrophil-to-lymphocyte ratio (NLR). [file Image_1.tif]
