# Supplementary material for: Clinical characteristics and prognostic factors of male breast cancer in China
Source: Front Oncol. 2024 Mar 8;14:1362826. doi: 10.3389/fonc.2024.1362826 (PMC10957788; doi:10.3389/fonc.2024.1362826)
Supplement: Supplementary file 2 [file Table_1.docx]

**Supplementary Table 1** Univariable Cox regression analysis for prognostic factors of MBC related to overall survival

|  | **HR (95%CI)** | ***P*-value** |  | **HR (95%CI)** | ***P*-value** |
| --- | --- | --- | --- | --- | --- |
| **Age(years)** | 1.11(1.07-1.16) | 4.347E-7 | **ER** |  |  |
| **Smoking history** |  |  | Negative | 1.00(reference) |  |
| No | 1.00(reference) |  | Positive | 0.31(0.11-0.92) | 0.035 |
| Yes | 1.26(0.59-2.68) | 0.548 | **PR** |  |  |
| **Drinking history** |  |  | Negative | 1.00(reference) |  |
| No | 1.00(reference) |  | Positive | 0.31(0.10-0.94) | 0.038 |
| Yes | 0.56(0.23-1.38) | 0.208 | **HER2** | |  |
| **Family history of cancer** | |  | Negative | 1.00(reference) |  |
| No | 1.00(reference) |  | Positive | 1.32(0.64-2.73) | 0.459 |
| Yes | 0.54(0.22-1.33) | 0.181 | **Type of surgery** | |  |
| **BMI (kg/m^2^)** |  |  | No surgery | 1.00(reference) |  |
| 18.5 - 25.0 | 1.00(reference) |  | Breast conserving surgery | 0.17(0.01-1.96) | 0.154 |
| <18.5 or ≥25.0 | 1.25(0.57-2.74) | 0.586 | Radical mastectomy | 0.17(0.04-0.75) | 0.019 |
| **Tumor size(cm)** |  |  | **Postoperative treatment** | |  |
| < 2.0 | 1.00(reference) |  | No | 1.00(reference) |  |
| ≥ 2.0 | 4.42(1.68-11.61) | 0.003 | Yes | 1.18(0.56-2.47) | 0.668 |
| **Differentiation** |  |  | **Distant metastasis** | |  |
| Well | 1.00(reference) |  | No | 1.00(reference) |  |
| Moderate | 1.21(0.35-4.15) | 0.760 | Yes | 4.56(2.17-9.58) | 6.200E-5 |
| Poor/  undifferentiated | 2.72(0.91-8.16) | 0.073 | **LMR** |  |  |
| **TNM stage** |  |  | < 3.67 | 1.00(reference) |  |
| I | 1.00(reference) |  | ≥ 3.67 | 0.38(0.17-0.86) | 0.020 |
| II | 2.03(0.62-6.61) | 0.240 | **PLR** |  |  |
| III | 3.12(0.96-10.17) | 0.059 | < 113.20 | 1.00(reference) |  |
| IV | 7.41(2.13-25.73) | 0.002 | ≥ 113.20 | 2.83(1.29-6.24) | 0.010 |
| **Lymph node metastasis** | |  | **NLR** |  |  |
| Negative | 1.00(reference) |  | < 2.13 | 1.00(reference) |  |
| Positive | 2.50(1.19-5.25) | 0.016 | ≥ 2.13 | 3.16(1.39-7.19) | 0.006 |
| **Ki67** |  |  |  |  |  |
| ≤15% | 1.00(reference) |  |  |  |  |
| >15% | 2.30(1.07-4.92) | 0.033 |  |  |  |

Abbreviation: BMI, body mass index; ER, estrogen receptor; PR, progesterone receptor; HER2, human epidermal growth factor receptor-2; LMR, lymphocyte-to-monocyte ratio; NLR, neutrophil-to-lymphocyte ratio; PLR, platelet-to-lymphocyte ratio.

**Supplementary Table 2** Univariable Cox regression analysis for prognostic factors of MBC related to disease-free survival^1^

|  | **HR (95%CI)** | ***P*-value** |  | **HR (95%CI)** | ***P*-value** |
| --- | --- | --- | --- | --- | --- |
| **Age(years)** | 1.06(1.02-1.09) | 0.001 | **Ki67** | |  |
| **Smoking history** |  |  | ≤15% | 1.00(reference) |  |
| No | 1.00(reference) |  | >15% | 2.74(1.41-5.35) | 0.003 |
| Yes | 1.12(0.55-2.27) | 0.761 | **ER** |  |  |
| **Drinking history** |  |  | Negative | 1.00(reference) |  |
| No | 1.00(reference) |  | Positive | 0.46(0.14-1.51) | 0.202 |
| Yes | 1.09(0.54-2.17) | 0.818 | **PR** |  |  |
| **Family history of cancer** | |  | Negative | 1.00(reference) |  |
| No | 1.00(reference) |  | Positive | 0.43(0.15-1.23) | 0.115 |
| Yes | 1.10(0.56-2.13) | 0.786 | **HER2** | |  |
| **BMI (kg/m^2^)** |  |  | Negative | 1.00(reference) |  |
| 18.5 - 25.0 | 1.00(reference) |  | Positive | 1.44(0.73-2.83) | 0.295 |
| <18.5 or ≥25.0 | 1.35(0.68-2.70) | 0.390 | **Postoperative treatment** | |  |
| **Tumor size(cm)** |  |  | No | 1.00(reference) |  |
| < 2.0 | 1.00(reference) |  | Yes | 1.17(0.61-2.25) | 0.628 |
| ≥ 2.0 | 3.19(1.57-6.47) | 0.001 | **LMR** |  |  |
| **Differentiation** |  |  | < 3.67 | 1.00(reference) |  |
| Well | 1.00(reference) |  | ≥ 3.67 | 0.50(0.26-0.96) | 0.037 |
| Moderate | 1.42(0.43-4.73) | 0.566 | **PLR** |  |  |
| Poor/  undifferentiated | 3.98(1.37-11.55) | 0.011 | < 113.20 | 1.00(reference) |  |
| **TNM stage** |  |  | ≥ 113.20 | 1.69(0.89-3.23) | 0.111 |
| I | 1.00(reference) |  | **NLR** |  |  |
| II | 1.56(0.69-3.53) | 0.289 | < 2.13 | 1.00(reference) |  |
| III | 1.72(0.61-4.84) | 0.304 | ≥ 2.13 | 1.09(0.56-2.12) | 0.793 |
| IV | 29.50(7.62-114.18) | 9.499E-7 |  |  |  |

Abbreviation: BMI, body mass index; ER, estrogen receptor; PR, progesterone receptor; HER2, human epidermal growth factor receptor-2; LMR, lymphocyte-to-monocyte ratio; NLR, neutrophil-to-lymphocyte ratio; PLR, platelet-to-lymphocyte ratio.

^1^only patients who did not develop distant metastases during the follow-up period were retained in this analysis.
